# Giant Baker Cyst Extending Up to the Gastrocnemius: A Case Report

**DOI:** 10.1055/s-0044-1787546

**Published:** 2024-06-12

**Authors:** Adeel Ahmed Siddiqui, Muhammad Waqas Khan, Taimoor Ali, Sajjad Ahmed, Shahzaeb Solangi, Javeria Qamar

**Affiliations:** 1 Departamento de Cirurgia Ortopédica, Dr. Ruth K. M. Pfau Civil Hospital, Karachi, Paquistão; 2 Dow Medical University of Health Sciences, Karachi, Paquistão

**Keywords:** cyst, gastrocnemius muscle, osteoarthritis, knee, synovial membrane

## Abstract

Baker cyst is an abnormal enlargement of the gastrocnemius-semimembranous bursa behind the knee joint due to an exit of joint fluid.

We herein report a rare case of giant Baker cyst in a rheumatic arthritic female patient. An MRI scan showed a complex, multiloculated cyst measuring 11.8 cm x 4.6 c, x 3.3 cm, arising from the left knee joint. Open surgical excision of the lesion was performed, and no recurrence was observed during the one-year follow up in the Rheumatology and Orthopedics Department.

A natural tendency towards intermuscular extension is observed; thus, intramuscular extensions are rarely reported in the literature. To the best of our knowledge, no Baker cyst of such size has been operated in a rheumatoid arthritis patient.

## Introduction

Baker cyst, an abnormal growth of the gastrocnemius-semimembranous bursa caused by the leakage of fluid from the knee joint into the bursa, is one of the most prevalent cystic lesions around the knee joint. 1 2 3 4 It may happen by normal movement of the fluid or synovial membrane herniation through a weak posterior knee capsule, whose damage may result from any direct or indirect trauma. 2 The frequently affected areas are the inferomedial or superficial layers of the knee joint, rarely spreading laterally or proximally. 1 3 It is a common pathological finding on sonography of the popliteal fossa, and 4.7% to 19% of magnetic resonance imaging (MRI) scans of the posterior knee masses show Baker cyst.

Naturally, the bursa tends to expand along the intermuscular planes or the spaces between the muscle and the joint capsule. Intramuscular extensions are rarely reported in the international literature, and not at all in Pakistan. We herein report a case of ruptured Baker cyst with an intraoperative extension in the gastrocnemius (GC) muscle.

## Case Report

A 35-year-old female patient with rheumatoid arthritis for past 5 years presented with a complaint of pain and swelling at the back of the left knee for 6 months. The pain was initially mild in intensity, aggravated by movement, and relieved with rest and analgesics. The swelling grew progressively, and the patient was initially managed with multiple aspirations in a local hospital, and negative fluid cultures were reported.

Upon examination, there was tender swelling measuring 10 cm x 5 cm at the left-sided popliteal fossa with intact, shiny overlying skin. The laboratory investigations included a complete blood profile, which showed a total leukocyte count (TLC) of 6.5 × 10 ^9^ /L, C-reactive protein of 82.0 mg/dL, and erythrocyte sedimentation rate of 33 mm/h.

A detailed report of the aspirated fluid indicated a yellowish turbid fluid with a specific gravity of 1.030, protein value of 4.48 g/dl, red blood cell (RBC) count of 4,500, and white blood cell (WBC) count of 28,480 microliters with 90% of polymorphonuclear (PMN) cells and 10% of mononuclear cells. The culture, however, showed no growth.

An ultrasound showed a bilobed, well-defined lesion to the hypoechoic area seen along the posterior aspect of the left knee, extending inferiorly up to the mid-alf and measuring 10.4 cm x3.5 cm. However, there was no evidence of vascularity on Doppler imaging. The popliteal vessels appeared unremarkable.

**Fig. 1:**
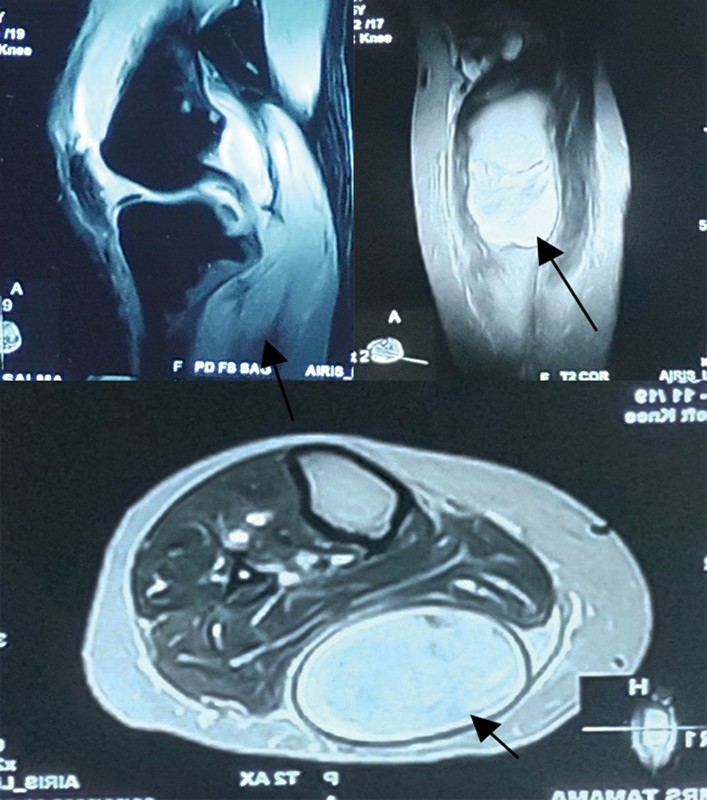
Magnetic resonance imaging (MRI) scan of the knee joint sequences showing a complex, multiloculated cyst with an internal hemorrhagic component arising from the posteromedial aspect of the knee joint and extending inferiorly into the calf region, causing compression over the posterior muscular compartment (upper left: sagittal section; right: coronal view; and bottom: axial view); all sections are slices > 2 mm, and T2-weighted images are shown, with all arrows pointing toward lesions in different views.

An MRI scan showed a complex, multiloculated cyst with an internal hemorrhagic component measuring 11.8 cm x4.6 cm x3.3 cm, arising from the posteromedial aspect of the left knee joint and extending inferiorly into the calf region, causing compression over the posterior muscular compartment. Its anteromedial margin appeared irregular. These were suggestive of a ruptured hemorrhagic Baker cyst. Mild to moderate joint effusion was noted as well ( Fig. 1 ).

**Fig. 2:**
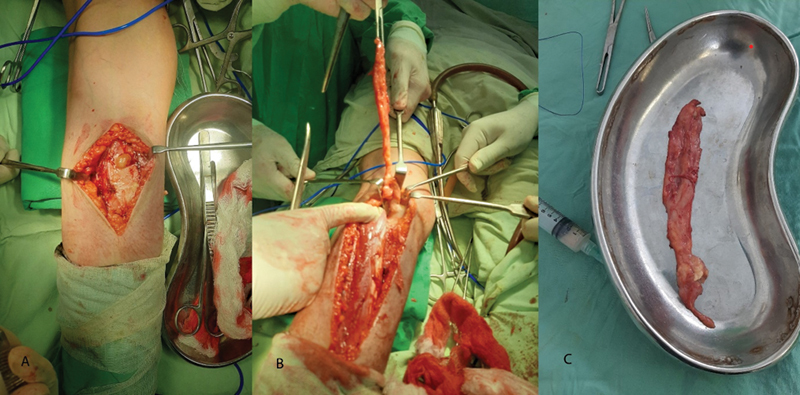
( **A,B** ) Longitudinal incision with cheese-like material coming out of the lesion, and the size of the excised cyst is compared to that of a standard kidney dish. One can appreciate excised cyst that was found intramuscularly in the gastrocnemius muscle. ( **C** ) Size comparison between the huge cyst and a standard kidney dish measuring 25 cm to 26 cm in length and 11 cm in width.

After all aseptic measures, preparation and draping were performed, a longitudinal incision was made at the left popliteal fossa, and subcutaneous tissue was dissected. A sac-like structure was found intramuscularly between the two heads of the GC muscles; it was dissected from the underlying muscle fibers, and a cheese-like material leaked from the cyst. The wound was closed in layers after proper irrigation. An aseptic dressing was applied, and the resected cyst was submitted to a histopathological analysis ( Fig. 2A–C ).

Microscopically, the cystic tissue fragments had fibrous walls with no definitive lining. There was chronic inflammatory infiltrate dominated by plasma cells, lymphocytes, histiocytes, and hemosiderin-laden cells. Together with fibrin and palisading histiocytes, fibrinoid necrosis was also observed; however, there was no evidence of malignancy or granuloma.

**Fig. 3:**
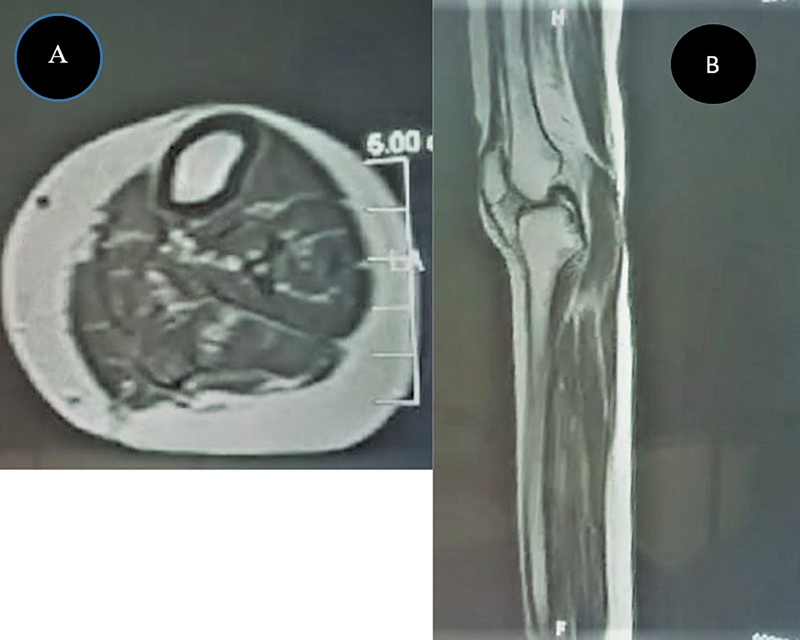
Postoperative MRI scan with contrast after open resection of a giant Baker cyst. ( **A** ) Axial view with absence of the lesion observed the in preoperative axial view; and ( **B** ) sagittal view of the knee with no residual cyst present.

A postoperative MRI scan with contrast showed complete excision of the lesion ( Fig. 3 ), and no complaints or recurrence were recorded in one year of follow up.

The present case report was approved by the institutional Review Board (ref: 04-2023); informed consent was obtained from the patient, and the purpose of the publication of the case report was briefed to patient.

## Discussion

Baker cyst, also known as a secondary popliteal cyst, is usually asymptomatic and observed on MRI scans when investigating other knee problems. It may present as a popliteal fossa mass, with or without pain, limited range of motion of the knee joint, and other compressive symptoms like thrombophlebitis, compartment syndrome, or entrapment neuropathy (either from the cyst or later, after its rupture). 4 5 6 7

We found eight cases of Baker cysts extending into the vastus medialis and medial head of the GC. 1 2 3 4 Fang et al., 1 in their case series, documented a Baker cyst that communicated to the joint via a narrow stalk between the GC and semimembranosus muscles and two additional cases of cysts within the GC muscle. In 2012, Kim et al. 2 reported a Baker cyst with intramuscular extension in the vastus medialis muscle through the weakened fascia, although there was no history of trauma. Moreover, the cyst was filled with hemorrhagic fluid.

Two similar cases were published by Li et al., 3 of two female patients who presented with knee pain and radiographic features of osteoarthritis. Effusion was detected via ultrasound, and later they were found to have Baker cyst extending into the medial head of the GC muscle.

Another intriguing case of posterior tibial neuropathy from a ruptured Baker cyst caused sole discomfort and paresthesia. Baker cyst is never examined in the differential diagnosis of individuals with calf and sole pain because it is rarely reported. 4

The current case is that of a female who presented with no symptoms of neuropathy or compression. The diagnosis of ruptured hemorrhagic Baker cyst was made through an MRI scan, and later turned out to be extending intramuscularly and involving the GC muscle on imaging; however, intramuscular extension was not identified.

In the literature, various conservative and operative interventions are mentioned. Conservative treatment options include aspirations, corticosteroid therapy, and methotrexate (an alternative for patients who are at high risk for surgery) injection intra-articularly and in the cysts. 8 Bandinelli et al. 9 reported satisfactory outcomes with ultrasound-guided direct steroid injection into a cyst and intra-articular steroid injection in situations identical to these; both techniques were deemed effective. In cases involving osteoarthritis, Acebes et al. 10 reported positive outcomes after the cyst contents were aspirated and corticosteroids were injected. Our results favored open excision because our patient was symptomatic and had multiple aspirations.

Although rare, lesions like a Baker cyst, primarily when ruptured, can involve the muscles around and below the knee joint. Lesions like these should be thoroughly investigated at the time of presentation rather than waiting for the appearance of compressive symptoms or neuropathy.
