# Supplementary material for: Giant Baker Cyst Extending Up to the Gastrocnemius: A Case Report
Source: Rev Bras Ortop (Sao Paulo). 2024 Jun 12;59(Suppl 2):e141–4. doi: 10.1055/s-0044-1787546 (PMC11679634; doi:10.1055/s-0044-1787546)
Supplement: Related texts

# Research article: Cisto de Baker gigante com extensão ao gastrocnêmio: Relato de caso

**Keywords:** cisto, membrana sinovial, músculo gastrocnêmio, osteoartrite do joelho

## Abstract

O cisto de Baker é um aumento anormal da bursa gastrocnêmio-semimembranosa atrás da articulação do joelho devido ao efluxo de fluido articular.

Relatamos um caso raro de cisto de Baker gigante em uma paciente com artrite reumatoide. A ressonância magnética mostrou um cisto complexo e multiloculado que media 11,8 cm x 4,6 cm x 3,3 cm, com origem na articulação do joelho esquerdo. Foi realizada excisão cirúrgica aberta da lesão. Não houve recidiva durante o acompanhamento de um ano no Departamento de Reumatologia e Ortopedia.

Há uma tendência natural de extensão intermuscular, de modo que os relatos de extensões intramusculares são raros na literatura. Pelo que sabemos, nenhum cisto de Baker desse tamanho foi operado em pacientes com artrite reumatoide.

## Introdução

O cisto de Baker, um crescimento anormal da bursa gastrocnêmio-semimembranosa causado pelo extravasamento de fluido da articulação do joelho para a bursa, é uma das lesões císticas mais prevalentes nessa articulação. 1 2 3 4 O cisto pode se desenvolver devido ao movimento normal do fluido ou a uma hérnia da membrana sinovial da articulação do joelho por enfraquecimento de sua cápsula posterior, que pode sofrer lesão decorrente de qualquer trauma direto ou indireto. 2 As áreas mais acometidas são as camadas inferomediais ou superficiais da articulação do joelho, com rara disseminação lateral ou proximal. 1 3 É um achado patológico comum na ultrassonografia da fossa poplítea. Entre 4,7% e 19% das ressonâncias magnéticas (RMs) de massas posteriores do joelho revelam cistos de Baker.

Naturalmente, a bursa tende a se expandir ao longo dos planos intermusculares ou dos espaços entre o músculo e a cápsula articular. Extensões intramusculares raramente são relatadas na literatura internacional, e nunca foram descritas no Paquistão. Apresentamos aqui um caso de rotura do cisto de Baker com extensão intraoperatória no músculo gastrocnêmio (GC).

## Relato de Caso

Uma paciente do sexo feminino, de 35 anos, com diagnóstico de artrite reumatoide havia 5 anos, apresentou queixa de dor e inchaço na região posterior do joelho esquerdo havia 6 meses. A princípio, a dor era de intensidade branda, agravada por movimento e aliviada com repouso e analgésicos. O inchaço aumentou de forma progressiva. A paciente foi inicialmente tratada com múltiplas aspirações em um hospital local, e as culturas de fluidos foram negativas.

O exame revelou um inchaço macio de 10 cm x 5 cm na fossa poplítea do lado esquerdo, com pele sobrejacente intacta e brilhante. Os exames laboratoriais incluíram hemograma completo. O número total de leucócitos foi de 6,5 × 10 ^9^ /L; os níveis de proteína C reativa foram de 82,0 mg/dL, e a velocidade de hemossedimentação foi de 33 mm/h.

O laudo detalhado do fluido aspirado indicou líquido turvo de cor amarelada com gravidade específica de 1,030, valor proteico de 4,48 g/dl, número de hemácias de 4.500 e número de leucócitos de 28.480 microlitros, com 90% de células polimorfonucleares neutrófilas e 10% de células mononucleares. Na cultura, porém, não houve crescimento.

A ultrassonografia mostrou uma lesão bilobada e bem definida na área hipoecoica ao longo da face posterior do joelho esquerdo, que se estendia inferiormente até o meio da panturrilha, e media 10,4 cm x 3,5 cm. No entanto, não havia evidências de vascularização na imagem em Doppler. Os vasos poplíteos tinham aparência normal.

A RM mostrou um cisto complexo, multiloculado, com componente hemorrágico interno e medindo 11,8 cm x 4,6 cm x 3,3 cm, com origem na face posteromedial da articulação do joelho esquerdo e extensão inferior até a região da panturrilha, que causava compressão sobre o compartimento muscular posterior. A margem anteromedial da lesão parecia irregular. Estes achados foram sugestivos da rotura de um cisto hemorrágico de Baker. Também foi observado derrame articular brando a moderado ( Fig. 1 ).

**Fig. 1:** Ressonância magnética das sequências da articulação do joelho mostrando um cisto complexo e multiloculado com um componente hemorrágico interno originando-se da face posteromedial da articulação e com extensão inferior até a região da panturrilha, que causava compressão sobre o compartimento muscular posterior (canto superior esquerdo: corte sagital; direito: corte coronal; e inferior: corte axial). Todos os cortes têm > 2 mm. A figura mostra imagens ponderadas em T2, e todas as setas indicam as lesões em diferentes projeções.

Depois de todas as medidas assépticas, preparo e colocação de campos cirúrgicos, foi feita uma incisão longitudinal na fossa poplítea esquerda, e o tecido subcutâneo foi dissecado. Uma estrutura semelhante a um saco foi encontrada intramuscularmente, entre as duas cabeças dos músculos GCs. Esta estrutura foi dissecada das fibras musculares subjacentes e houve extravasamento de um material de aparência caseosa do cisto. A ferida foi fechada em camadas após a irrigação adequada. Foi aplicado um curativo asséptico e o cisto ressecado foi submetido à análise histopatológica ( Fig. 2A–C ).

**Fig. 2:** ( **A,B** ) A incisão longitudinal mostra o material de aparência caseosa saindo da lesão e o tamanho do cisto excisado é comparado ao de uma cuba rim padrão. Note que o cisto excisado estava em posição intramuscular no músculo gastrocnêmio. ( **C** ) Comparação do enorme cisto com uma cuba rim padrão de 25 cm a 26 cm de comprimento e 11 cm de largura.

Microscopicamente, os fragmentos de tecido cístico apresentavam paredes fibrosas sem revestimento definitivo. Havia um infiltrado inflamatório crônico dominado por plasmócitos, linfócitos, histiócitos e macrófagos com hemossiderina. Juntamente com fibrina e histiócitos em paliçada, também foi observada necrose fibrinoide, mas sem evidências de malignidade ou granuloma.

Depois, uma RM pós-operatória com contraste revelou a excisão completa da lesão ( Fig. 3 ). Não houve queixa ou recidiva em um ano de acompanhamento.

**Fig. 3:** Ressonância magnética contrastada pós-operatória da paciente após a ressecção aberta de um cisto gigante de Baker. ( **A** ) Corte axial com ausência da lesão observada no corte axial pré-operatório; e ( **B** ) corte sagital do joelho, evidenciando a ausência de cisto residual.

Este relato de caso foi aprovado pelo Comitê de Ética em Pesquisa institucional sob número 04-2023. A paciente assinou o termo de consentimento livre e esclarecido e foi informada sobre o objetivo de publicação do relato de caso.

## Discussão

O cisto de Baker, também conhecido como cisto poplíteo secundário, é geralmente assintomático e revelado por uma RM para investigação de outros problemas no joelho. Pode apresentar-se como uma massa na fossa poplítea, acompanhada ou não de dor, limitação da amplitude de movimento da articulação do joelho e outros sintomas compressivos, como tromboflebite, síndrome compartimental ou neuropatia por encarceramento (decorrente do cisto ou posterior à sua ruptura). 4 5 6 7

Encontramos oito casos de cistos de Baker com extensão até o vasto medial e a cabeça medial do GC. 1 2 3 4 Em uma série de casos, Fang et al. 1 relataram um cisto de Baker que se comunicava com a articulação por meio de uma haste estreita entre os músculos GC e semimembranoso, e dois casos de cistos no músculo GC. Em 2012, Kim et al. 2 relataram um cisto de Baker com extensão intramuscular no músculo vasto medial através da fáscia enfraquecida, apesar da ausência de histórico de trauma. Além disso, o cisto estava cheio de fluido hemorrágico.

Dois casos semelhantes foram publicados por Li et al., 3 de duas mulheres que apresentavam dor no joelho e características radiográficas de osteoartrite. A efusão foi detectada por ultrassom e, posteriormente, descobriu-se em ambas as pacientes a presença de um cisto de Baker que se estendia até a cabeça medial do músculo GC.

Outro caso intrigante de neuropatia tibial posterior decorrente da rotura de um cisto de Baker causou desconforto na planta do pé e parestesia. O cisto de Baker nunca é incluído no diagnóstico diferencial de indivíduos com dor na panturrilha e na planta do pé porque é raramente relatado. 4

O caso aqui relatado é o de uma mulher que não apresentava sintomas de neuropatia ou compressão. O diagnóstico de rotura do cisto hemorrágico de Baker foi feito por RM e, depois, descobriu-se que o cisto se estendia por via intramuscular e acometia o músculo GC; no entanto, a extensão intramuscular não foi identificada.

A literatura menciona diversas intervenções conservadoras e cirúrgicas. As opções de tratamento conservador são por aspirações, administração de corticosteroides e injeção intra-articular e intracística de metotrexato (uma alternativa em pacientes com alto risco cirúrgico). 8 Bandinelli et al. 9 documentaram desfechos satisfatórios com injeção direta de corticosteroides guiada por ultrassonografia em um cisto e a injeção intra-articular de corticosteroides em situações idênticas a estas; as duas técnicas foram consideradas eficazes. Nos casos de osteoartrite, Acebes et al. 10 relataram desfechos positivos após a aspiração do conteúdo do cisto e a injeção de corticosteroides. Nossos resultados favoreceram a excisão aberta porque nossa paciente era sintomática e tinha sido submetida a múltiplas aspirações.

Embora raras, lesões como o cisto de Baker, principalmente em casos de rotura, podem acometer os músculos em volta e abaixo da articulação do joelho. Lesões como essas devem ser minuciosamente investigadas no momento da apresentação, em vez de se aguardar o surgimento de sintomas compressivos ou neuropatia.
